# TCDD Induces the Hypoxia-Inducible Factor (HIF)-1α Regulatory Pathway in Human Trophoblastic JAR Cells

**DOI:** 10.3390/ijms151017733

**Published:** 2014-09-30

**Authors:** Tien-Ling Liao, Su-Chee Chen, Chii-Reuy Tzeng, Shu-Huei Kao

**Affiliations:** 1 Graduate Institute of Medical Science, College of Medicine, Taipei Medical University, Taipei 110, Taiwan; E-Mail: pneumoniae0909@gmail.com; 2 School of Medical Laboratory Science and Biotechnology, College of Medical Science and Technology, Taipei Medical University, Taipei 110, Taiwan; 3 Department of Obstetrics and Gynecology, Cathay General Hospital, Taipei 110, Taiwan; E-Mail: chanduki49@gmail.com; 4 Center for Reproductive Medicine & Sciences; Taipei Medical University Hospital, Taipei 110, Taiwan; E-Mail: tzengcr@tmu.edu.tw; 5 Department of Obstetrics and Gynecology, Taipei Medical University Hospital, Taipei 110, Taiwan

**Keywords:** dioxin, trophoblastic cell, hypoxia induction factor-1α, reactive oxygen species

## Abstract

The exposure to dioxin can compromise pregnancy outcomes and increase the risk of preterm births. 2,3,7,8-Tetrachlorodibenzo-*p*-dioxin (TCDD) has been demonstrated to induce placental hypoxia at the end of pregnancy in a rat model, and hypoxia has been suggested to be the cause of abnormal trophoblast differentiation and placental insufficiency syndromes. In this study, we demonstrate that the non-hypoxic stimulation of human trophoblastic cells by TCDD strongly increased hypoxia inducible factor-1 alpha (HIF-1α) stabilization. TCDD exposure induced the generation of reactive oxygen species (ROS) and nitric oxide. TCDD-induced HIF-1α stabilization and Akt phosphorylation was inhibited by pretreatment with wortmannin (a phosphatidylinositol 3-kinase (PI3K) inhibitor) or *N*-acetylcysteine (a ROS scavenger). The augmented HIF-1α stabilization by TCDD occurred via the ROS-dependent activation of the PI3K/Akt pathway. Additionally, a significant increase in invasion and metallomatrix protease-9 activity was found in TCDD-treated cells. The gene expression of vascular endothelial growth factor and placental growth factor was induced upon TCDD stimulation, whereas the protein levels of peroxisome proliferator-activated receptor γ (PPARγ), PPARγ coactivator-1α, mitochondrial transcription factor, and uncoupling protein 2 were decreased. Our results indicate that an activated HIF-1α pathway, elicited oxidative stress, and induced metabolic stress contribute to TCDD-induced trophoblastic toxicity. These findings may provide molecular insight into the TCDD-induced impairment of trophoblast function and placental development.

## 1. Introduction

The appropriate development of the placenta is crucial to normal fetal programming [1]. The placenta, a highly vascularized organ, develops to facilitate nutrient uptake, waste removal, and gas exchange between mother and fetus. Many endocrine disruptors and environmental toxicants are prone to accumulation in the placenta, negatively affecting fetal development. The endocrine disruptor 2,3,7,8-tetrachlorodibenzo-*p*-dioxin (TCDD), commonly known as the most toxic dioxin, has been shown to disrupt hormone signaling, leading to perturbed ovarian steroidogenesis, placental vascular remodeling [2,3,4], and increased spontaneous abortion in humans and animals [5]. Indeed, the *in utero* exposure to TCDD causes placenta toxicity, stillbirth, and early neonatal death in many species [6,7,8]. In a retrospective cohort study of women who resided in highly contaminated areas with TCDD exposure, the effect of TCDD was strongly associated with reduced birth weight and gestational age [9].

Convincing evidence from animal studies has shown that TCDD affects the placental vascular network during embryo and fetal development by altering blood flow and vascular morphology in the placental labyrinth zone [3,7,10,11]. In a rat model, TCDD-exposed placentas were found to be in a hypoxic state at the end of pregnancy [12]. The increased incidence of fetal death after exposure to TCDD is suggested to be due to placental hypoxia. In response to hypoxia, hypoxia-inducible factor-1 alpha (HIF-1α) is a critical regulator in adaptation to hypoxia. Under normoxic conditions, the cellular HIF-1α level is regulated by prolyl hydroxylases (PHDs), leading to its ubiquitylation and proteasomal degradation. However, PHD activity is inactivated due to either a lack of oxygen or exposure to various chemical inhibitors, leading to HIF-1α stabilization [13]. Placental vascular development can be affected via activation of the HIF-1α pathway and its targets, such as vascular endothelial growth factor (VEGF), leading to altered placental vascularization [3].

As the molecular mechanisms behind TCDD-induced HIF-1α stabilization remain to be characterized, we identified the molecular signaling of TCDD-induced HIF-1α stabilization in the trophoblastic JAR cell line. The data from this study demonstrate that augmented HIF-1α stabilization by TCDD occurred via the reactive oxygen species (ROS)-dependent activation of the phosphoinositide-3-kinase (PI3K)/Akt pathway. The molecular targets of TCDD, including the up-regulation of *VEGF* and *placenta* *growth factor* (*PlGF*) expression and the suppression of peroxisome proliferator-activated receptor γ (PPARγ) and PPARγ coactivator-1α (PGC-1α), were revealed in the TCDD-treated trophoblastic cells. These results indicate that TCDD exposure may activate the HIF-1α pathway, induce oxidative stress, and impair bioenergetic homeostasis, leading to the perturbation of trophoblastic cell function and the placental vascular network.

## 2. Results

### 2.1. Induced Hypoxia-Inducible Factor-1 Alpha (HIF-1α) Stabilization in 2,3,7,8-Tetrachlorodibenzo-p-dioxin (TCDD)-Treated Human Trophoblastic Cells

**Figure 1:**
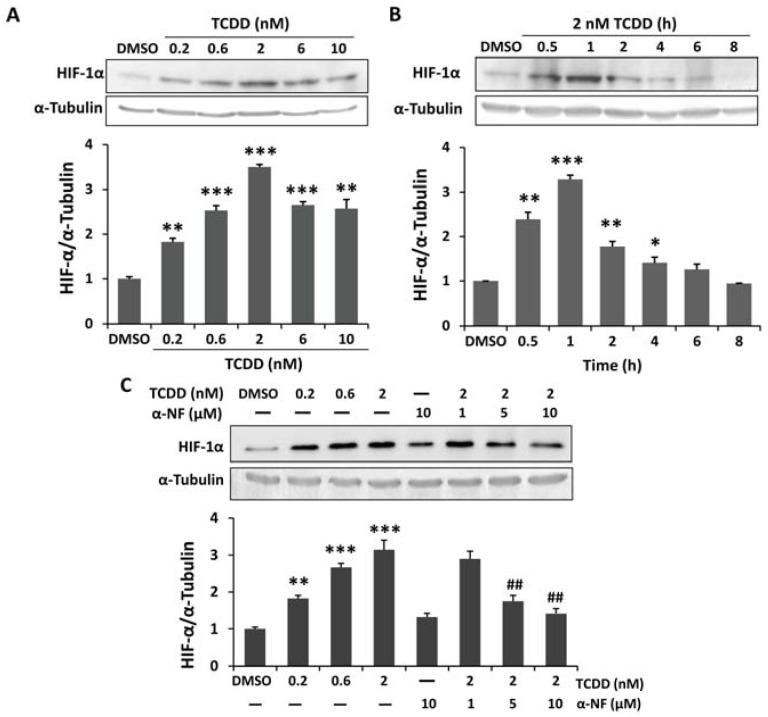
Dose- and time-dependent effects of 2,3,7,8-Tetrachlorodibenzo-*p*-dioxin (TCDD) on hypoxia-inducible factor-1 alpha (HIF-1α) stabilization in human trophoblastic cells. Western blots of a representative experiment were shown. (**A**) Human trophoblastic cells were treated with various doses of TCDD (0.2, 0.6, 2, 6, and 10 nM) for 1 h and immunodetected with an HIF-1α specific antibody; (**B**) Cells were incubated with 2 nM TCDD for various time periods (0.5–8 h); (**C**) Cells were pretreated with α-naphthoflavone (α-NF) (Aryl hydrocarbon receptor (AhR) antagonist) for 24 h. TCDD-induced HIF-1α stabilization was attenuated by pretreatment of the cells with α-NF (1, 5, and 10 μM). The mean densitometry data from independent experiments were normalized to the results obtained for cells in the absence of TCDD (as control group). DMSO (0.1% *v*/*v*) alone was used as the solvent control. The plots are presented as the mean ± standard deviation (SD) (*n* = 3). * *p* < 0.05; ** *p* < 0.01; *** *p* < 0.001 compared with the control group. ## *p* < 0.01 compared with the 2 nM TCDD-treated group.

In the present study, the JAR cell line was used to address the effects of TCDD; this cell line was verified to maintain some of the trophoblast-specific characteristics of primary trophoblasts. We applied 0.2–10 nM of TCDD, which was equivalent to a high human exposure level, to evaluate the cytoeffects on the trophoblastic cells. To identify whether the exposure to TCDD induces hypoxia in cells, we treated the trophoblastic cells with various doses and cultured for different periods of time. As shown in Figure 1A,B, the stabilization of HIF-1α proteins was significantly increased by 3.29 ± 0.16-fold in the cells exposed to 2 nM TCDD for 1 h relative to the control group (0.1% DMSO group). Aryl hydrocarbon receptor (AhR) is a known dioxin receptor, and the data showed that the TCDD-induced up-regulation of HIF-1α proteins was attenuated by pretreatment of the cells with α-naphthoflavone (α-NF), an AhR antagonist (Figure 1C). This demonstrated that TCDD-induced HIF-1α stabilization occurred via the AhR pathway.

### 2.2. Enhanced Reactive Oxygen Species (ROS) and Nitric Oxide (NO) Generation in TCDD-Treated Human Trophoblastic Cells

**Figure 2:**
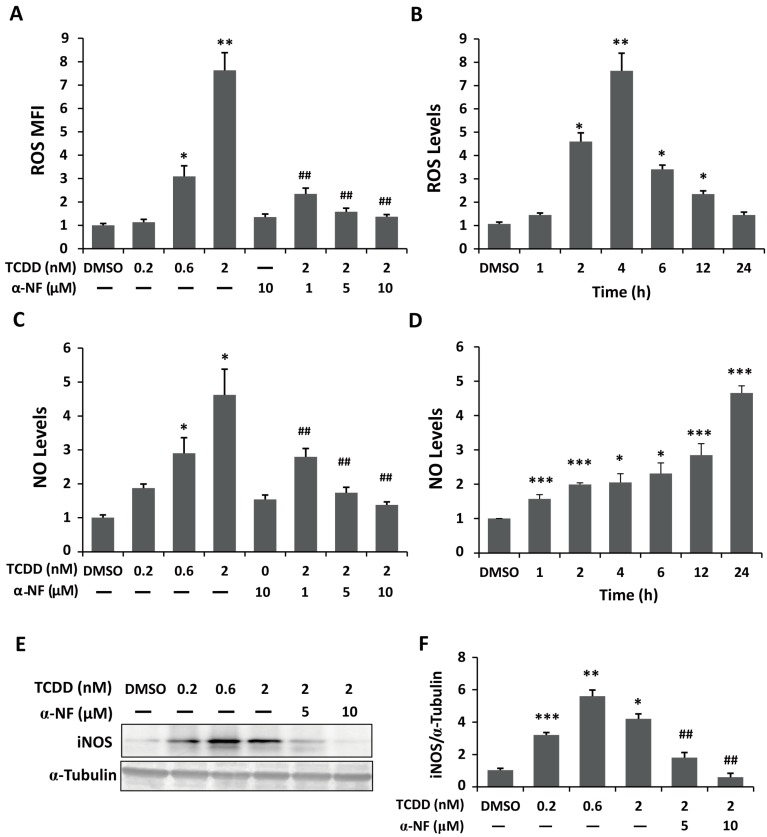
TCDD-mediated generation of reactive oxygen species (ROS) and nitric oxide (NO) in trophoblastic cells. (**A**,**C**) Cells were treated with the various doses of TCDD (0.2, 0.6, 2, 6, and 10 nM) with or without α-NF pretreatment (the AhR antagonist); (**B**,**D**) Cells were treated with 2 nM TCDD for different time periods; (**A**,**B**) ROS were measured through 5-(and-6)-chloromethyl-2',7'-dichlorodihydrofluorescein diacetate acetyl ester (H_2_DCFDA) staining followed by flow cytometry; (**C**,**D**) NO production was detected using the Griess Reagent, as described in Methods; (**E**) The expression level of inducible NOS (iNOS) protein in the TCDD-treated cells with or without α-NF pretreatment was detected by western blotting and quantified by densitometry; (**F**) Pretreatment with α-NF reduced TCDD-induced ROS generation, NO production, and iNOS expression. The plots are presented as the mean ± SD (*n* = 3). * *p* < 0.05; ** *p* < 0.01; *** *p* < 0.001 compared with the control group. ## *p* < 0.01 compared with the 2 nM TCDD-treated group.

To determine whether TCDD exposure induces the generation of reactive oxygen species (ROS) and nitric oxide (NO), we measured H_2_O_2_ production by performing 5-(and-6)-chloromethyl-2',7'-dichlorodihydrofluorescein diacetate acetyl ester (CM-H_2_DCFDA) staining and NO levels by the Griess method. Dose-responsive increases in ROS and NO levels were found in the TCDD-treated cells (Figure 2A,C). After exposure to 2 nM TCDD, the production of hydrogen peroxide and nitrite/nitrate increased nearly 7.6- and 4.6-fold, respectively, compared with the control group (Figure 2A,C). The highest stimulation of ROS generation was found with the treatment of 2 nM TCDD for 4 h (Figure 2B). A time-dependent induction of NO production is shown in Figure 2D. The TCDD-induced generation of ROS and NO was attenuated by pretreatment of the cells with α-NF (Figure 2A,C). In addition, TCDD stimulated the expression of inducible nitric oxide synthase (iNOS) as well as NO production in the trophoblastic cells (Figure 2E,F).

### 2.3. Phosphoinositide-3-Kinase and Akt Signaling Are Involved in TCDD-Induced Hypoxia and ROS Generation in Trophoblastic Cells

**Figure 3:**
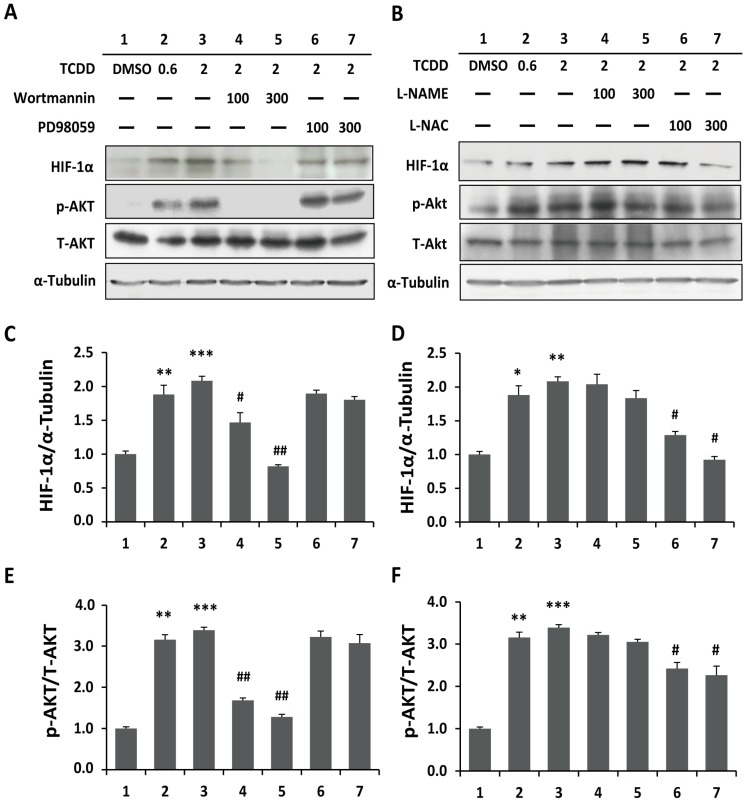
ROS signaling and phosphoinositide-3-kinase (PI3K) pathway are involved in TCDD-induced HIF-1α stabilization and Akt phosphorylation. (**A**) Cells were pre-treated with wortmannin (PI3K inhibitor) or PD98059 (2'-amino-3'-methoxyflavone, the mitogen-activated protein kinase kinase (MEK) inhibitor) for 30 min and then incubated with 2 nM TCDD for 1 h; (**B**) Cells were pre-treated with *N*-acetylcysteine (NAC, ROS scavenger) or *N*^ω^-nitro-l-arginine methyl ester hydrochloride (NAME, NO scavenger) for 30 min and then incubated with 2 nM TCDD for 1 h. The protein levels of HIF-1α, phospho-Akt (p-Akt), and total Akt (T-Akt) were immunodetected by western blotting; (**C**–**F**) The mean densitometry data from independent experiments were normalized to the results obtained for cells in the absence of TCDD (as control group). The plots are presented as the mean ± SD (*n* = 3). * *p* < 0.05; ** *p* < 0.01; *** *p* < 0.001 compared with the control group. # *p* < 0.05; ## *p* < 0.01 compared with the 2 nM TCDD-treated group.

To identify whether the trophoblastic cells respond to TCDD-induced HIF-1α stabilization via PI3K and Akt signaling, the cells were pretreated with wortmannin (PI3K inhibitor) or PD98059 (2'-amino-3'-methoxyflavone, the mitogen-activated protein kinase kinase (MEK) inhibitor), and the protein levels of HIF-1α, phospho-Akt (p-Akt at Serine 473), and total Akt (T-Akt) were immunodetected. The PI3K inhibitor wortmannin significantly reduced the levels of HIF-1α stabilization and Akt phosphorylation in a dose-dependent manner. In contrast, no significant effects were found with PD98059 pretreatment (Figure 3A,C,E).

We subsequently determined whether ROS and NO signaling is involved in HIF-1α accumulation and Akt phosphorylation in TCDD-treated trophoblastic cells. The antioxidant *N*-acetylcysteine (NAC, ROS scavenger) or *N*^ω^-nitro-l-arginine methyl ester hydrochloride (NAME, an inhibitor of nitric oxide synthase) was applied to measure the molecular effects. NAC but not NAME significantly reduced HIF-1α stabilization and Akt phosphorylation (Figure 3B,D,F), supporting a regulatory mechanism of ROS signaling in TCDD-induced HIF-1α accumulation and Akt phosphorylation.

### 2.4. Induced Cell Invasion and Activation of Matrix Metalloproteinase in TCDD-Treated Trophoblastic Cells

**Figure 4:**
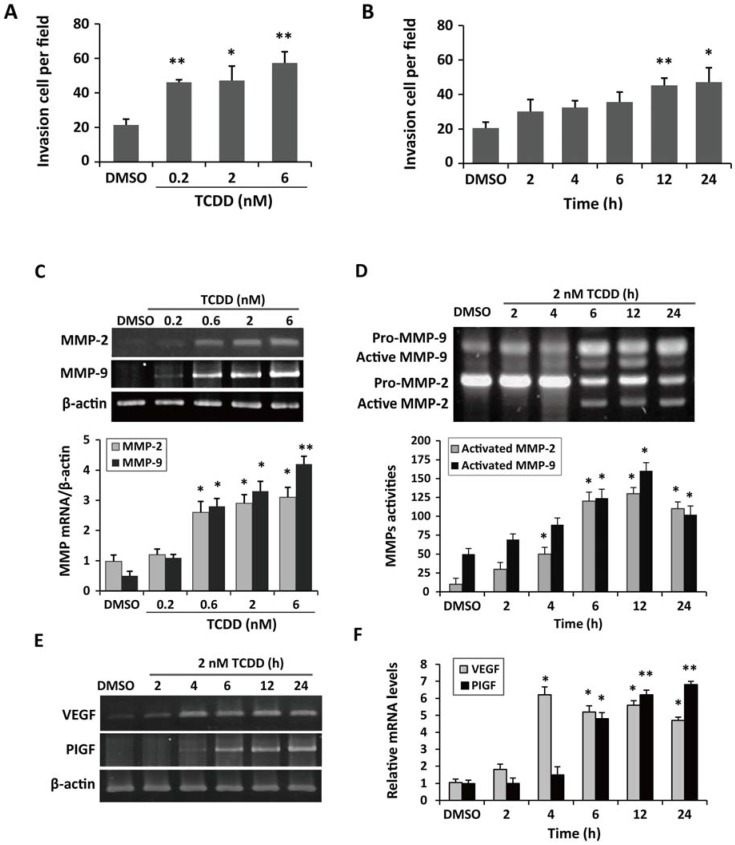
TCDD induced gene expression during cell invasion and vascularization. (**A**) Cells were treated with various concentrations of TCDD (0, 0.2, 0.6, 2, and 6 nM) for 24 h; (**B**) Cells were treated with 2 nM TCDD for different periods of time (0, 2, 4, 6, 12, and 24 h). The invading trophoblastic cells were collected and counted using a Matrigel invasion assay. Enhanced cell invasion occurred in a dose- and time-dependent manner; (**C**,**D**) Increased mRNA expression levels of *matrix metalloproteinase* (*MMP*)-*2* and *MMP*-*9* were revealed by a semi-quantitative RT-PCR analysis; (**D**) The increased active forms of MMP-9 and MMP-2 were detected in the cells treated with TCDD for 24 h by gelatin zymography; (**E**) Electrophoretogram of the RT-PCR products of vascular endothelial growth factor (VEGF) and placenta growth factor (PlGF) amplified from TCDD-treated trophoblast cells; (**F**) Increased *VEGF* and *PlGF* mRNA levels were revealed by real-time quantitative PCR. The plots are the mean ± S.D. (*n* = 3). * *p* < 0.05 compared with each control group; ** *p* < 0.01 compared with each control group.

We performed a Matrigel invasion assay, western blotting, and a gelatin zymography assay to investigate whether TCDD exposure induces cell invasion, *matrix metalloproteinase* (*MMP*) expression, and MMP activation. As shown in Figure 4A,B, TCDD induced cell invasion in a dose- and time-dependent manner, along with a dose-responsive increase in *MMP* gene expression by real-time quantitative PCR. A time-dependent sequential activation of MMP-9 and MMP-2 was also found by gelatin zymography assays (Figure 4D). We furthermore examined two growth factors, VEGF and PlGF, both of which are members of the VEGF family and involved in modulation of placental angiogenesis and vascular remodeling. The time-dependent up-regulation of *VEGF* and *PlGF* gene expression was found in the TCDD-treated cells (Figure 4E,F), indicating that the exposure of TCDD may alter the placental vascular network and placental function.

### 2.5. Inhibition of Peroxisome Proliferator-Activated Receptor γ (PPARγ) and PPARγ Coactivator-1α (PGC-1α) Expression in TCDD-Treated Trophoblastic Cells

**Figure 5:**
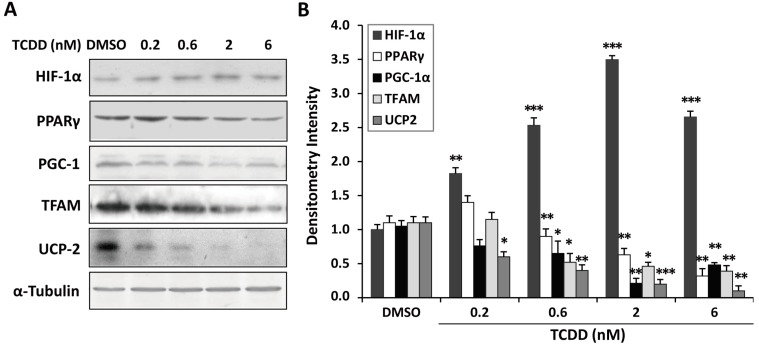
Peroxisome proliferator-activated receptor γ (PPARγ) and PPARγ coactivator-1α (PGC-1α) are targets of TCDD. (**A**) The expression profiles of *HIF-1α*, *uncoupling protein 2* (*UCP2*), *PPARγ*, *PGC-1α*, and mitochondrial transcription factor (TFAM) were assessed by western blotting; (**B**) The protein levels were quantified by densitometry. Reduced expression of *PPARγ*, *PGC1α*, *TFAM*, and *UCP2* was found in the TCDD-treated trophoblastic cells. The plots are the mean ± S.D. (*n* = 3). * *p* < 0.05; ** *p* < 0.01; *** *p* < 0.001 compared with each control group.

**Figure 6:**
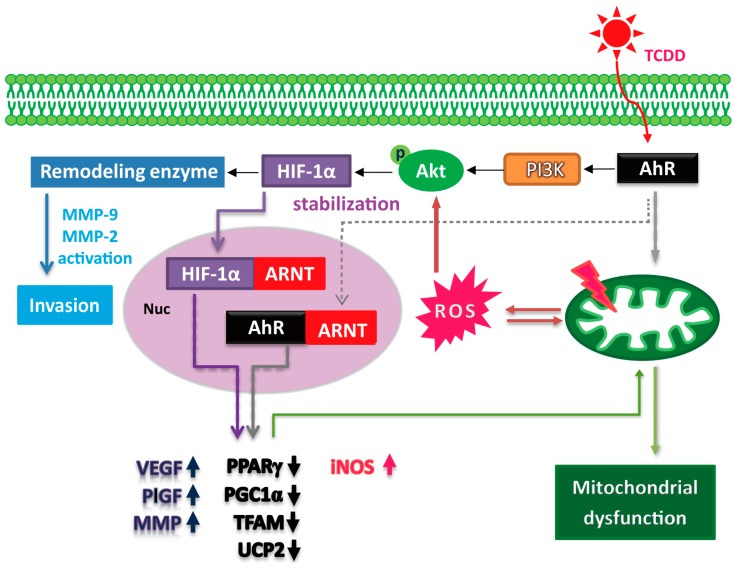
Schematic diagram of the TCDD induced HIF-1α molecular signaling. TCDD induced HIF-1α stabilization via the AhR or ROS-dependent activation of the PI3K/Akt pathway. A significant increase in invasion, MMP9 activity, and *VEGF* and *PlGF* gene expression was found in the TCDD-treated cells. Furthermore, down regulation of metabolic regulators PPARγ and PGC-1α was involved in the TCDD-induced insults. TFAM and UCP2, downstream targets of PGC1α, were concurrently suppressed and may lead to mitochondrial dysfunction. Taken together, our results indicate that an activated HIF-1α pathway, elicited oxidative stress, and induced metabolic stress contribute to TCDD-induced trophoblastic toxicity.

PPARγ and its coactivator PGC-1α have been demonstrated to be essential for energy metabolism, trophoblast differentiation, and placentogenesis. To investigate the effect of TCDD treatment on *PPARγ* and *PGC-1α* expression, we examined protein levels by western blotting. TCDD treatment significantly suppressed the expression levels of *PPARγ* and *PGC1α* (Figure 5A,B) and reduced the expression levels of *mitochondrial transcription factor* (*TFAM*), one of the downstream transcription factors of PGC-1α targets. Concurrently, uncoupling protein 2 (UCP2), which may be another effector downstream of PGC-1 and contributes to protection against oxidative damage, was decreased in the TCDD-treated cells. These results indicated that TCDD impaired mitochondrial bioenergetics, leading to mitochondrial dysfunction and altered metabolic homeostasis. Our results indicate that an activated HIF-1α pathway, elicited oxidative stress, and induced metabolic stress contribute to TCDD-induced trophoblastic toxicity (Figure 6).

## 3. Discussion

Dioxins bioaccumulate and are persistently present in environmental pollutants. Once inside the human body, dioxins become stored in fat [14]. Along with the perinatal exposure to TCDD, placental tissue is the highest deposition site compared to other fetal tissues [15]. Dioxins can cross the placenta, exposing the developing embryo and fetus and adversely affecting placental and fetal development [15,16]. Dioxins have been demonstrated to exert their effects by binding to a specific cellular protein known as AhR. In our study, the exposure to TCDD activated HIF-1 signaling in trophoblastic cells and an AhR antagonist (α-NF) attenuated the TCDD-induced HIF-1α stabilization. On the other hand, Nie’s group has reported that both the AhR and hypoxia pathways require aryl hydrocarbon receptor nuclear translocator (ARNT) for transcriptional activation [17]. Taken together these observations, it suggests that there is a cross-talk between AhR and hypoxia transcriptional pathways.

Due to the limitations of investigating the effects of TCDD on human pregnancy, the trophoblastic JAR cell line was instead used in the study. The JAR cells were generated from gestational choriocarcinomas and have been frequently used as models for in vitro studies of trophoblasts [18]. JAR cells have been proven adequate for studying the human trophoblast differentiation and function, but they may not completely represent normal trophoblasts. During placentation, a delicate and complicated niche is built by the cross-talk between trophoblasts and stromal cells, which involves various hormones, growth factors, and cytokines [19]. The communication of trophoblasts and stromal cells may impact the toxic insults of TCDD and need to be further investigated. In addition, a previous study conducted by Fukushima’s group did not show an increase in HIF-1α or VEGF in the TCDD-exposed HTR-8/SVneo cells [20]. HTR-8/SVneo cell lines were derived from first trimester placental villous explants via transformation with Simian Virus-40 large T antigen transformation and are often deemed as having stem-like characteristics [21]. We suggested that the differential response to TCDD exposure of HTR-8/SVneo cells may be due to its stem-like characteristics harboring higher basal levels of HIF-1α. The discrepancy in responses to TCDD in JAR cells and HTR-8/SVneo cells should be further investigated.

HIF-1 is a heterodimeric transcription factor that regulates the transcriptional activation of several genes responsive to hypoxia. During normoxia, HIF-1α is propyl-hydroxylated and binds with von-Hippel-Lindau (VHL complex), leading to the proteasome-mediated degradation of HIF-1α [22]. With the loss of VHL or during hypoxic conditions, HIF-1α accumulates and forms a heterodimer with ARNT and then translocates to the nucleus and binds to HIF-responsive elements (HREs), leading to the transcription of target genes such as angiogenic factor VEGF. The up-regulation of HIF-1α signaling leading to placental vascular defects and half the size of the labyrinth layer was observed in a VHL-null placenta [23].

In spite of oxygen tensions, the HIF-1α protein has been demonstrated to be up-regulated under normoxia in response to growth factors, hormones, cytokines, and environmental chemicals [24,25,26]. Accumulating evidence has shown that HIF-1 can be induced by non-hypoxic factors in a redox-sensitive manner [13,27]. ROS have been implicated as oxygen sensors and linked to the regulation of HIF-1. Thus, ROS are suggested to play a critical role in HIF-1 activation under non-hypoxic conditions. In this study, we found that TCDD induced ROS generation, and the supplement of *N*-acetylcysteine (a ROS scavenger) was found to reduce TCDD-induced HIF-1α stabilization. This finding indicates that ROS signaling contributes to TCDD-induced HIF-1α stabilization.

Placental development is a highly complicated process involving interactions between maternal and fetal tissues. Oxygen tension is key regulator for the establishment of maternal-placental circulation [28,29]. The human placenta affords a unique environment in terms of oxygenation, as it undergoes a transition from a low to a more oxygenated environment [30], and this physiological switch in oxygen tension and dynamic HIF-α signaling is required for placental development. In humans, two peaks of HIF-α protein levels are found at 7–10 weeks (the first trimester) and at 14–18 weeks of gestation (the second trimester). Outside these two peaks, the placenta was shown to have lower levels of HIF-α. Our data showed that TCDD induces HIF-1α stabilization partly through ROS signaling. However, further investigation is necessary to determine whether TCDD exposure can disrupt VHL or prolyl hydroxylase domain (PHD) activity, resulting in enhanced HIF-1α stabilization.

TCDD was demonstrated to alter gene expression in a direction that promotes migration and invasion processes in several cell types [31,32,33]. Our data also showed that TCDD significantly induced trophoblastic cell invasion and MMP activation. Different from cancer with its destructive and disorganized invasion, trophoblast invasion is precisely controlled by uterine constituents including decidual cells and immune cells [34]. Both cells govern the uteroplacental junction to restrict excessive trophoblast invasion [34]. In a pre-eclamptic rat model, a deeper trophoblast invasion was found in a hypertensive rat than that in a normal rat [35]. Moreover, under experimental hypoxia, the low oxygen tensions caused abnormal invasion, with reduced integrin α1, human leukocyte antigen-G (HLA-G), and vascular endothelial-cadherin expression, resulted in abnormal invasion and inadequate vascular transformation [29,36]. The precise mechanism and effects of TCDD-enhanced trophoblast invasion needs further investigation.

Placental development requires adequate and organized interaction of vascular growth factors and their receptors, including VEGF, PlGF, and VEGFR-1 (fms-like tyrosine kinase, Flt-1) or VEGFR-2 (kinase insert domain-containing region, KDR). Both VEGF and PlGF, acting through VEGFRs, have been implicated in playing a pivotal role in placental vascular development [26,37]. Our findings showed higher stimulations of *VEGF* and *PIGF* gene expression in TCDD-treated trophoblastic cells. The up-regulation of *VEGF* and *PIGF* gene expression has also been demonstrated in the TCDD-exposed pregnant rat [3]. TCDD differentially regulates the expression of angiogenic factors, which in turn may alter placental vascular remodeling and result in fetal growth abnormalities. A study has found that in response to hypoxia and ischemia, *VEGF* and *VEGFR*-*2* expression was increased in villous blood vessels, whereas *PlGF* and *VEGFR*-*1* were induced in villous trophoblasts [37]. However, some studies have shown that under hypoxia, villous trophoblast expressed significantly higher levels of *VEGF* mRNA and *VEGF* proteins, but lower levels of *PlGF* mRNA and protein were found [38,39]. These controversial results may be due to the differential temporal and spatial expression of *PlGF* and need to be further clarified.

Our data showed that TCDD treatment significantly suppressed the expression levels of *PPARγ* and *PGC*-*1α*. PPARγ is a ligand-activated transcription factor and plays essential roles in placental development by controlling trophoblast differentiation and invasion and by regulating the interface vascular exchange with maternal blood [40]. PPARγ-null murine embryos die at midgestation because of abnormalities in all placental layers, with a small labyrinth and expanded giant cell layer [41]. Moreover, PPARγ is a transcription factor target and partner of PGC-1α, which activates many nuclear receptors that in turn regulate mitochondrial biogenesis in various tissues, gluconeogenesis in the liver, and thermogenesis in brown adipose tissue [42]. PGC-1α is proposed to be a powerful regulator of angiogenesis, an important process for developing a healthy placenta and embryo in normal pregnancy [43]. Recently, TCDD has been identified as binding to PPARα and PPARγ, leading to the disruption of granulosa cell function and a decrease in estradiol synthesis [44]. PGC1α is also a target of TCDD. In hepatic cells, TCDD was found to increase the acetylation and ubiquitin-dependent proteasomal degradation of PGC1α, resulting in decreased PGC1α target gene expression [45]; TFAM and UCP2, downstream targets of PGC1α, were concurrently suppressed. Rather than protect cells from oxidative damage, UCP2 was shown to engage in the maintenance of placental capacity. A significant decrease in placental UCP2 expression was found in small for gestational age (SGA) newborns compared to appropriate for gestational age (AGA) newborns [46]. In our previous study, we demonstrated that TCDD causes mitochondrial genome instability, mitochondrial dysfunction, and cell death [47]. In addition, HIF-1 was demonstrated to be linked to the control of a series of molecular mechanisms involved in energy and redox homeostasis. Additionally, mitochondrial dysfunction was found to promote HIF-1α stabilization via ROS signaling [48,49]. Taken together, these results indicate that TCDD impairs mitochondrial biogenesis, bioenergetics, and metabolic capacity via PPARγ and PGC-1α signaling.

## 4. Experimental Section

### 4.1. Reagents

Fetal calf serum (FCS) was obtained from Biochrome KG (Berlin, Germany). Culture flasks and plates were purchased from Corning (Corning, NY, USA) and precoated with 1.6 µg/cm^2^ of Vitrogen 100^®^ (Celtrix Lab, Palo Alto, CA, USA) before cell seeding. RPMI 1640 was purchased from Sigma-Aldrich (St. Louis, MO, USA). *N*-acetylcysteine (NAC), *N*-nitro-l-arginine-methyl ester (NAME), and other tissue culture reagents were purchased from Sigma (St. Louis, MO, USA). The PI-3-kinase inhibitors wortmannin and ERK1/2 inhibitor PD98059 were obtained from Calbiochem (La Jolla, CA, USA). CM-H_2_DCFDA and Griess Reagent (G-7921) were purchased from Molecular Probes (Eugene, OR, USA).

### 4.2. Cell Culture

The JAR cell line (ATCC number: HTB-144™) was derived from a human trophoblastic tumor of the placenta and was performed according to the ethical guideline and approved by the institutional ethical committee. Cells were maintained at 37 °C in humidified 5% CO_2_ in RPMI 1640 medium with 2 mM l-glutamine and containing 1.5 g/L sodium bicarbonate, 4.5 g/L glucose, 10 mM *N*-2-hydroxyethylpiperazine-*N*'-2-ethanesulfonic acid (HEPES), and 1.0 mM sodium pyruvate, supplemented with 10% fetal calf serum. Different concentrations of TCDD (Sigma-Aldrich, St. Louis, MO, USA) were added to the trophoblastic cells to reach final concentrations of 0.2, 0.6, 2, and 6 nM; 0.1% DMSO was used as the control group.

### 4.3. Western Blots

Whole-cell lysates were either used directly from TCDD-treated samples or prepared separately in whole-cell extract buffer (50 mM Tris, pH 7.4, 150 mM NaCl, 5 mM EDTA, 0.1% SDS, 1 mM phenazine methosulfate, and complete protease inhibitor; Roche Molecular Biochemicals, Basel, Switzerland). Equal amounts of protein were electrophoresed through an acrylamide gel, transferred to polyvinylidene difluoride membranes (Millipore, Billerica, MA, USA), and immunoblotted according to standard protocols using 5% nonfat dry milk in Tris-buffered saline with 0.1% Tween 20. Anti-HIF-1α (1:2000 at dilution), -Akt (1:2000 at dilution), -p-Akt (Ser473, 1:2000 at dilution), -PGC-1α (1:2000 at dilution), -PPARγ (1:2000 at dilution), and -α-tubulin (1:2000 at dilution) (Santa Cruz Biotechnology, Santa Cruz, CA, USA) and secondary antibodies, anti-mouse IgG conjugated alkaline phosphatase (Santa Cruz Biotechnology) or anti-rabbit IgG conjugated horseradish peroxidase (Cell Signaling Technologies, Beverly, MA, USA) were used, followed by enhanced chemiluminescence detection (Amersham Biosciences, Uppsala, Sweden).

### 4.4. Flow Cytometry Analysis for ROS Generation

Aliquots of 1 × 10^6^ cells were gently stained with H2DCFDA (Molecular Probes, Eugene, OR, USA) in the dark for 15 min at room temperature. After staining, the cells were washed with phosphate buffered saline (PBS) and applied for flow cytometric analysis. All analyses were performed with a FACScan (Becton Dickson, San Jose, CA, USA) and measured at 490–500 nm excitation and 525 nm emission. A minimum of 30,000 cells per sample were analyzed. Data were acquired in the list mode, and the relative proportions of cells within different areas of the fluorescence profile were quantified using the LYSYS II software program (Becton Dickson, Franklin Lakes, NJ, USA).

### 4.5. Measurement of Nitrite by the Griess Reagent

The levels of NO were measured by assaying the culture supernatants for NO_2_^−^, a stable product of NO with molecular oxygen. The assay was performed with the Griess Reagent kit from Molecular Probes. The Griess Reagent kit (G-7921, Molecular Probes, Eugene, OR, USA) was prepared by mixing equal volumes of 0.5% *N*-(1-naphthyl)ethylenediamine dihydrochloride and 0.5% sulfanilic acid immediately prior to the experiments. For the determination of nitrite, 100 µL of supernatant was mixed with 50 µL of Griess Reagent and incubated at room temperature in the dark for 30 min. The absorbance was measured at 548 nm, and the nitrite concentrations were calculated from a standard curve obtained using standards containing increasing concentrations of NaNO_2_ at 0, 1, 5, 10, and 25 µM.

### 4.6. RNA Extraction and Real-Time Quantitative PCR

Total RNA was extracted with an RNeasy Mini Kit (Qiagen, Valencia, CA, USA) according to the manufacturer’s instructions. First-strand cDNA synthesis was performed with 5 Units of Moloney Murine Leukemia Virus (MMLV) reverse transcriptase (Epicentre, Madison, WI, USA), 1 μg RNA, and 50 picomoles of random primers (Promega, Madison, WI, USA). cDNA was PCR-amplified under the following conditions: denaturation at 94 °C for 5 min, 30 cycles of 40 s at 94 °C, 40 s at 51 °C, and 2 min 30 s at 72 °C, and a final elongation at 72 °C for 5 min. The primer sequences were as follows: *MMP-2-F*, 5'-AGGATCATTGGCTACACACC-3'; *MMP-2-R*, 5'-AGCTGTCATAGGATGTGCCC-3'; MMP-9-F, 5'-CGCAGACATCGTCATCCAGT-3'; *MMP-9-R*, 5'-GGATTGGCCTTGGAAGATGA-3'. Real-time PCR was performed using a Roche LightCycler apparatus (Roche Diagnostics GmbH, Manheim, Germany) with the Roche LightCycler FastStart DNA Master SYBR Green kit. The reactions were performed as follows: an initial denaturation at 95 °C for 300 s, followed by 40 cycles of 1 s at 95 °C, 6 s at 58 °C, and 18 s at 72 °C. The threshold cycle number of the *β-actin* and *OPA1* genes was determined for each individual quantitative PCR run. The primer sequences for qPCR were as follows: *VEGF-qF*, 5'-CGAAACCATGAACTTTCTGA-3'; *VEGF-qR*, 5'-CCTCAGTGGGCACACACTCC-3'; *PlGF-qF*, 5'-CAGAGGTGGAAGTGGTACCCTTCC-3'; *PlGF-qR*, 5'-CGGATCTTTAGGAGCTGCATGGTGAC-3'; *β-actin-qF*, 5'-CCAACCGCGAGAAGATGA-3'; *β-actin-qR*, 5'-CCAGAGGCGTACAGGGATAG-3'. The amplification efficiency for each tested gene was normalized to β-actin.

### 4.7. Cell Invasion Assay

For the invasion assay, the 24-well Matrigel-coated chemotaxis Boyden chambers were used (BD Falcon, Le Pont de Claix, France). The chambers were assembled using 8-μm pore size transwell-inserts as the upper chamber and 24-well plates as lower chambers. Cell culture inserts were coated with 100 µL Matrigel (1:1 diluted with PBS). Aliquots of 1 × 10^5^ JAR cells were placed in the upper chamber in presence of 0.2, 2, or 6 nM TCDD for 2, 4, 6, 12, or 24 h. At the end of incubation times, cells that traversed the Matrigel to the lower surface of the insert were fixed with 10% formalin, stained with crystal violet and counted under a light microscope.

### 4.8. Gelatin Zymography

MMP activity in cell lysates was determined by gelatin zymography. Cell lysate protein concentrations were determined by the BCA protein assay (Thermo Scientific, Rockford, IL, USA). Equal amounts of cell protein or equal volumes of supernatant were separated under nonreducing conditions in the presence of SDS on a 10% zymogram gel containing 0.1% gelatin (Invitrogen, Grand Island, NY, USA). The gels were incubated for 90 min in 2.7% Triton X-100 to remove the MMP activity-inhibiting SDS and then overnight in zymogram developing buffer (5 mM Tris, 4 mM HCl, 20 mM NaCl, 0.5 mM CaCl_2_, and 0.002% Brij 35). The gels were stained with Coomassie blue (Bio-Rad, Hercules, CA, USA) for 3 h and destained with a Coomassie destain mix until clear bands were visible on the dark-blue background.

### 4.9. Statistical Analysis

Data are expressed as the mean ± standard deviation (S.D.). The data were assessed using the *t*-test, and *p* < 0.05 was considered statistically significant.

## 5. Conclusions

In summary, we found that TCDD induced HIF-1α stabilization via the ROS-dependent activation of the PI3K/Akt pathway. The supplement of *N*-acetylcysteine (a ROS scavenger) was found to reduce this TCDD-induced HIF-1α stabilization and Akt phosphorylation. A significant increase in invasion, MMP9 activity, and *VEGF* and *PlGF* gene expression was found in the TCDD-treated cells. Furthermore, critical metabolic regulators PPARγ and PGC-1α were involved in the TCDD-induced insults. It has been asserted that exposure to TCDD via induced HIF-1α stabilization increases the risk of abnormal trophoblast differentiation and function and is the basis of many placenta-based pregnancy disorders, including pre-eclampsia and fetal growth restriction. These findings that TCDD induces hypoxia and oxidative stress in trophoblastic cells may provide molecular insight into human placental pathologies.

## Supplementary Materials

Supplementary Figure can be found at http://www.mdpi.com/1422-0067/15/10/17733/s1.

## Supplementary Files

Supplementary File 1
