# Supplementary material for: TCDD Induces the Hypoxia-Inducible Factor (HIF)-1α Regulatory Pathway in Human Trophoblastic JAR Cells
Source: Int J Mol Sci. 2014 Sep 30;15(10):17733–50. doi: 10.3390/ijms151017733 (PMC4227186; doi:10.3390/ijms151017733)

Supplementary Information

Figure S1. PD98059 treatment inhibited the TCDD-induced MEK phosphorylation. Cells were pre-treated with PD98059 (MAPK kinase (MEK) inhibitor) for 30 min and then incubated with 2 nM TCDD for 1 h. PD98059 significantly reduced the levels of MEK phosphorylation in a dose-dependent manner. No significant inhibitory effects on the TCDD-induced HIF-1α stabilization were found with PD98059 pretreatment.

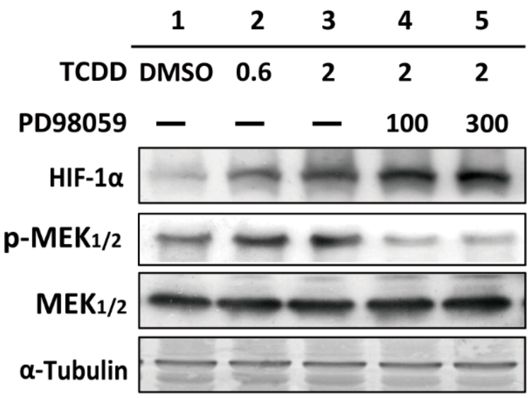

Supplement: Supplementary File 1 [file ijms-15-17733-s001.pdf]
